# Altered binaural hearing in pre-ataxic and ataxic mutation carriers of spinocerebellar ataxia type 3

**DOI:** 10.1007/s12311-023-01519-3

**Published:** 2023-01-30

**Authors:** Heike Jacobi, Martin Andermann, Jennifer Faber, Felicitas Baumann, André Rupp

**Affiliations:** 1 Department of Neurology, Heidelberg University Hospital, Im Neuenheimer Feld 400, 69120 Heidelberg, Germany; 2 Department of Neurology, Bonn University Hospital, Bonn, Germany; 3 German Center for Neurodegenerative Diseases, Bonn, Germany; 4 Section of Biomagnetism, Department of Neurology, Heidelberg University Hospital, Im Neuenheimer Feld 400, 69120 Heidelberg, Germany

**Keywords:** Binaural hearing, Psychoacoustics, MEG, Spinocerebellar ataxia

## Abstract

Brainstem degeneration is a prominent feature of spinocerebellar ataxia type 3 (SCA3), involving structures that execute binaural synchronization with microsecond precision. As a consequence, auditory processing may deteriorate during the course of disease. We tested whether the binaural “Huggins pitch” effect is suitable to study the temporal precision of brainstem functioning in SCA3 mutation carriers. We expected that they would have difficulties perceiving Huggins pitch at high frequencies, and that they would show attenuated neuromagnetic responses to Huggins pitch. The upper limit of Huggins pitch perception was psychoacoustically determined in 18 pre-ataxic and ataxic SCA3 mutation carriers and in 18 age-matched healthy controls. Moreover, the cortical N100 response following Huggins pitch onset was acquired by means of magnetoencephalography (MEG). MEG recordings were analyzed using dipole source modeling and comprised a monaural pitch condition and a no-pitch condition with simple binaural correlation changes. Compared with age-matched controls, ataxic but not pre-ataxic SCA3 mutation carriers had significantly lower frequency limits up to which Huggins pitch could be heard. Listeners with lower frequency limits also showed diminished MEG responses to Huggins pitch, but not in the two control conditions. Huggins pitch is a promising tool to assess brainstem functioning in ataxic SCA3 patients. Future studies should refine the psychophysiological setup to capture possible performance decrements also in pre-ataxic mutation carriers. Longitudinal observations will be needed to prove the potential of the assessment of Huggins pitch as a biomarker to track brainstem functioning during the disease course in SCA3.

## Introduction

Spinocerebellar ataxias (SCAs) are a heterogeneous group of autosomal dominantly inherited progressive ataxia disorders. The most common subtype worldwide is spinocerebellar ataxia type 3 (SCA3) [1]. It is characterized by great neuropathological complexity and affects various regions of the nervous system, especially the cerebellum and the brainstem as well as basal ganglia, thalamus, spinal cord, dorsal root ganglia, and sensory peripheral nerves [2]. Ataxia often starts in the fourth decade of life, but onset age is highly variable, partly depending on the repeat length of the expanded allele [3, 4]. As SCA3 is caused by CAG trinucleotide repeat expansions in the *ATXN3* gene with full penetrance, pre-ataxic mutation carriers provide the unique opportunity to study early pathophysiological changes.

Recent studies have confirmed that regional brain tissue loss in SCA3 starts before ataxia onset especially in the brainstem, cerebellum, and spinal cord [5, 6]. Among the different parts of the brain subjected to morphometric analysis, pons-related alterations show the largest effect size (*d’* = 1.03). Importantly, since pons houses the nuclei of the olive complex, the integrity of brainstem auditory pathways may be also limited early in the course of the disease [7, 8]. Moreover, pathoanatomical studies have demonstrated that auditory brainstem nuclei as well as the respective fiber tracts (lateral lemniscus, trapezoid body) are affected in SCA2, SCA3, and SCA7 patients [7]. In particular, the medial superior olive (MSO) is known to encode interaural time differences (ITDs) with exquisite resolution of about 10 µs, corresponding to an angle difference of about 1°. Notable is the fact that the MSO input is driven by the largest, fastest, and temporally most accurate synapses of the mammalian brain [9]. Any systemic pathophysiological impact leads to reduced temporal precision in ITD encoding [10]. Zeigelboim et al. detected auditory processing impairments in patients with SCA (including SCA3) using the staggered spondaic word and the random gap detection test, which points to difficulties in binaural temporal resolution and integration; moreover, decreased performance in binaural hearing tasks has also been described in pre-ataxic and ataxic SCA13 mutation carriers who showed no obvious impairment in monaural pure-tone sensitivity [11]. Together, temporal precision renders binaural hearing a valid model to study the integrity of central neural processing, and even subtle jitter in binaural input synchronization can induce psychoacoustic and neurophysiological performance decrements. [12–14]

A simple and intriguing tool to study binaural processing is the so-called Huggins pitch phenomenon [15]. When a white noise is presented diotically, listeners hear it as a simple noise without any pitch; however, if a continuous phase shift (2π) is imposed across a narrow frequency band at one of the two ears, a faint pitch—the Huggins pitch—can be perceived within the noise. The height of the pitch increases when the phase-shifted band moves towards higher frequency regions, thereby requiring better (i.e., temporally more accurate) synchronization of the binaural input. In a recent investigation, Rupp et al. studied *N* = 72 listeners from 8 to 84 years and found a strong association between listener’s age and the ability to perceive Huggins pitch. The limit up to which Huggins pitch was heard decreased with increasing age (*r* = − 0.79***). Similarly, magnetoencephalography (MEG) recordings in a sub-group of *N* = 21 listeners revealed that the magnitude of the cortical pitch response to Huggins pitch onset was negatively correlated with age (*r* = − 0.45*) while responses to diotic pitches were not correlated with age (*r* = − 0.08 n.s.). In addition to Huggins pitch, the neuromagnetic activity evoked by (overall) interaural correlation changes in broadband noise also showed smaller magnitudes with increasing age (*r* = − 0.52*). The fact that these effects occur only with binaural but not monaural stimuli points to a key role of the superior olive in the brainstem for temporally precise input synchronization; moreover, performance in binaural listening tasks is not necessarily correlated with audiometric status [16]. This is in line with animal studies suggesting that temporal processing limitations are likely caused by neurodegeneration of the brainstem, whereas “audiometric” hearing impairment is typically due to peripheral origin [17, 18]. Binaural stimuli like Huggins pitch might thus represent powerful tools for the assessment of temporal processing in the central nervous system, in particular the olive complex of the pons.

The present study was designed to test the applicability of Huggins pitch in the psychophysiological assessment of SCA3 mutation carriers. In light of Rupp et al.’s results, and based on Faber et al.’s findings regarding changes in the pontine volume of SCA3 mutation carriers, we hypothesized that these individuals would show reduced upper limits of Huggins pitch in psychoacoustic tasks, and that they would exhibit smaller neuromagnetic responses to Huggins pitch onset and to broadband interaural correlation changes. Furthermore, given the binaural specificity of the above-described effects, we expected that diotic pitch stimuli would not lead to comparable psychoacoustic or neuromagnetic response decrements in SCA3 mutation carriers.

## Patients and Methods

### Participants

Pre-ataxic and ataxic SCA3 mutation carriers (*N* = 18) were enrolled between July 2019 and February 2022. The presence and severity of ataxia was assessed using the Scale for the Assessment and Rating of Ataxia (SARA); clinically manifest ataxia was defined by a SARA score ≥ 3 [19]. The examination of neurological signs other than ataxia was done using the Inventory of Non-Ataxia Signs (INAS) [20]. The presence and severity of depressive symptoms was assessed with a Rasch-based depression screening (DESC) [21]. Further, the Montreal Cognitive Assessment (MoCA) was applied to screen for cognitive impairment. [22]

The control group comprised *N* = 18 healthy adults and was carefully matched for age. None of the participants reported any history of hearing impairment or psychiatric or neurological disorders.

Written informed consent was obtained from every subject and they were not paid for participation. The experimental procedures were conducted in accordance with the ethical standards as laid down in the 1964 Declaration of Helsinki and its later amendments and approved by the local ethics committee (Medical Faculty, University of Heidelberg, S-242/2019).

### Psychoacoustic Task

A 3-alternative forced choice task [23] was conducted, with two runs per listener, to determine the frequency up to which Huggins pitch could be heard by the individual participants. Each trial consisted of three 800-ms-long broadband Gaussian noise bursts (bandpass-filtered at 10–10.000 Hz and ramped on/off with 20 ms cosine ramps; inter-stimulus interval: 500 ms) that were presented to the listeners in random order. In one of the noises, a 2π phase transition was imposed in the right-ear channel to elicit Huggins pitch, and subjects were asked to identify this stimulus from the three noise bursts. The bandwidth of the phase transition was set to 10% of the Huggins pitch frequency (HP-*f*_0_). The HP-*f*_0_ starting value was set to 400 Hz; HP-*f*_0_ increased after correct responses in two successive trials, and it decreased after one incorrect response. The HP-*f*_0_ change factor for an increase or decrease was set to 1.5 and was set to 0.75 after two reversals. The upper frequency limit of Huggins pitch was determined as the mean HP-*f*_0_ of the last 6 reversals in the task. The task and all sounds for the MEG experiment described below were built using MATLAB 7.1 (The Mathworks, Inc. Natick, MA). Stimuli were presented at 65 dB SPL and with 48,000 Hz sampling rate, using Beyerdynamic DT 770 PRO headphones attached to an external digital-analog converter and a headphone amplifier (RME ADI 2-DAC FS). To avoid fine structure cues due to frozen noise, each single noise was generated freshly from running noise. HP-*f*_0_ differences between groups were statistically analyzed using the Wilcoxon-test.

### MEG Stimulation and Recordings

The MEG experiment comprised two binaural and one monaural conditions which were recorded in separate blocks, each block with a total duration of 15 min. In each block, stimuli were played 200 times, with inter-stimulus intervals randomly distributed between 800 and 900 ms. Within conditions, each single stimulus segment had a length of 750 ms and was equipped with 50-ms Hanning windows at its onset and offset. All sounds were generated using in-house MATLAB scripts, converted by a 24-bit sound card (RME ADI 8DS AD/DA interface), attenuated by Tucker Davis Technologies PA-5, and delivered to the listeners via shielded Etymotic Research (ER3) earphones, attached to 90-cm plastic tubes and foam inserts. The earphones were driven by a Tucker Davis Technologies HB-7 headphone buffer.

Besides the Huggins pitch condition, there was also a binaural condition in which the interaural correlation of two successive broadband Gaussian noise bursts changed from + 1 to − 1, or vice versa, with square-rooted 10-ms Hanning ramp crossfading to avoid monaural cues at the transition. Changing the interaural correlation induces a diffuse change in the perceived spatial location of the noise, but not a pitch percept. The monaural condition was made of iterated rippled noise (IRN) [24] which is built by adding the copy of a noise back to the original signal with a given time delay. IRN pitch corresponds to the reciprocal of the time delay, and pitch salience increases with the number of iterations. In this study, we employed IRN with 20 iterations. In the IRN and Huggins pitch conditions, stimuli were assembled to short triplets, in an effort to separate the neuromagnetic energy onset response (EOR) from pitch onset (POR) and pitch change responses (PCR) (e.g., Andermann et al. 2021) [25]. Each triplet began with a noise burst without pitch, followed by two stimuli with different pitch. The first pitch corresponded to 880 Hz, and the second pitch was either a perfect fifth up or down, relative to the first pitch.

Neuromagnetic field gradients in response to the stimulation were acquired using a Neuromag-122 MEG gradiometer system (Elekta Neuromag Oy, Finland) [26] inside a shielded room (IMEDCO, Hägendorf, Switzerland). Data were sampled at 1000 Hz and low-pass filtered at 330 Hz. Prior to the recordings, fiducials and 100 surface points were digitized using a Polhemus 3D-Space Isotrack2 system to determine the head shape and position relative to the MEG gradiometers. During the recordings, participants watched a silent movie of their own choice to maintain stable vigilance, and they were asked to ignore the sounds in the earphones.

### MEG Data Analysis

Gradiometer data were analyzed using the BESA 5.2. software (BESA GmbH, Gräfelfing, Germany), with a spherical head model and a homogeneous volume conductor. After removing noisy channels, epochs with amplitudes > ± 8000 fT/cm and gradients > ± 800 fT/cm/ms were automatically removed using the BESA rejection tool. Across all conditions, on average, 85.9% (SD = 10.4) of the sweeps remained in the analysis. Prior to spatio-temporal source analysis [27, 28], data were zero-phase filtered at 1–30 Hz. For all conditions, neuromagnetic responses to the transitions between the noise segments were pooled, and a source model with one equivalent dipole per hemisphere was fitted on the prominent N100m POR in the IRN condition, with the fit interval centered about 30–50 ms around its peak. No constraints were applied for fitting, but symmetry was applied to stabilize the fit when necessary. The source models were then applied as spatio-temporal filters to derive the source waveforms within the unfiltered IRN, Huggins pitch, and interaural correlation conditions; here, principal component analysis [29] based on the last 100 ms of the epochs was used for drift compensation. The resulting source waveforms were exported to MATLAB for further graphical and statistical analysis; similarly, dipole coordinates were exported in approximate Talairach space. [30]

N100m peak amplitudes were extracted by using the grand-average N100m peak latency as a reference and averaging the individual waveform in a 50-ms window around this value, separately for every participant. This procedure ensured that amplitude values could be determined for each single listener even when the waveform did not show a prominent peak response (which was the case for a few listeners in the Huggins pitch condition). For between-group comparison of the N100m amplitude, the non-parametric Wilcoxon test was chosen from the R statistical package (Version 4.1.0). Correlations were computed using the Spearman rank correlation coefficients.

### Data Sharing

Neurophysiological data is available in OSF (https://osf.io/rmshp/).

## Results

### Patient Characteristics

Demographic and clinical characteristics of the SCA3 mutation carriers are summarized in Table 1. 18 SCA3 mutation carriers (9 males, 9 females; mean age = 43.56 ± 12.54 years) and 18 age-matched healthy volunteers (11 males; 7 females; mean age = 42.23 ± 11.78 years) were enrolled. Six of the SCA3 mutation carriers were pre-ataxic defined by a SARA score < 3 (mutation carriers: mean age = 32.26 ± 5.63 years; matched controls: mean age = 32.01 ± 5.70 years). There were no differences with respect to the repeat length of the expanded or normal allele, DESC scores, or MoCA scores. However, ataxic SCA3 mutation carriers were older_,_ and had higher SARA scores, as well as higher INAS counts (Table 1).

**Table 1:** Demographic and clinical characteristics of the SCA3 mutation carriers

	SCA3 total	SCA3 pre-ataxic	SCA3 ataxic	Wilcoxon-test
Number of participants	18	6	12	n.a
Men/women (*n*)	9/9	3/3	6/6	n.a
Age	43.6 ± 12.5	32.3 ± 5.6	49.2 ± 11.1	*W* = 47, *p* = 0.001
Short allel	25.3 ± 11.3	25.3 ± 2.6	25.3 ± 13.7	*W* = 10, *p* = 0.239
Long allel	68.7 ± 3.9	69.5 ± 4.1	68.3 ± 3.9	*W* = 32.5, *p* = 0.775
SARA	10.1 ± 8.5	1.1 ± 0.7	14.5 ± 6.7	*W* = 72, *p* = 0.001
INAS count	3.4 ± 2.7	1.0 ± 0.9	4.6 ± 2.6	*W* = 67, *p* = 0.004
DESC	8.4 ± 8.9	5.8 ± 8.4	9.8 ± 9.1	*W* = 49, *p* = 0.238
MoCA	27.7 ± 2.1	28.5 ± 1.2	27.3 ± 2.3	*W* = 24, *p* = 0.273

*SARA* Scale for the Assessment and Rating of Ataxia, *INAS* Inventory of Non-Ataxia Signs, *DESC* Rasch-based depression screening, *MOCA* Montreal Cognitive Assessment

Data are given as mean ± standard deviation

### Psychoacoustic Results

**Fig. 1:**
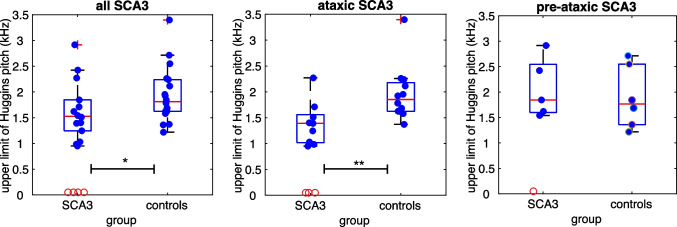
The boxplots show the upper frequency limit of Huggins pitch for all SCA3 mutation carriers and age-matched controls (left panel), ataxic SCA3 mutation carriers (middle panel), and pre-ataxic SCA3 mutation carriers (right panel). Four SCA3 mutation carriers were not able to perceive Huggins pitch at all

There were clear differences among the participants regarding Huggins pitch perception. In contrast to healthy controls, three out of 12 ataxic patients as well as one pre-ataxic mutation carrier were not able to perceive Huggins pitch *at all* (*χ*^2^(1) = 4.50, *p* = 0.034). When the median upper frequency limit of Huggins pitch was compared between groups (see Fig. 1), it was found to be significantly lower in SCA3 mutation carriers than in age-matched controls (*Z* = 1.65, *p* = 0.0492, *d*’ = 0.55). Among the SCA3 mutation carriers, ataxic patients had much lower cut-off frequencies than age-matched controls (*Z* = 2.45, *p* = 0.0071; *d*’ = 1.20), whereas in pre-ataxic SCA3 mutation carriers, results were similar to the control group (*Z* = 0.46, *p* = 0.3289, *d*’ = − 0.29).

### MEG Results

The results presented below are based on *N* = 14 SCA3 mutation carriers; in four cases, no MEG data could be acquired due to non-removable metal parts as a contraindication for MEG.

**Fig. 2:**
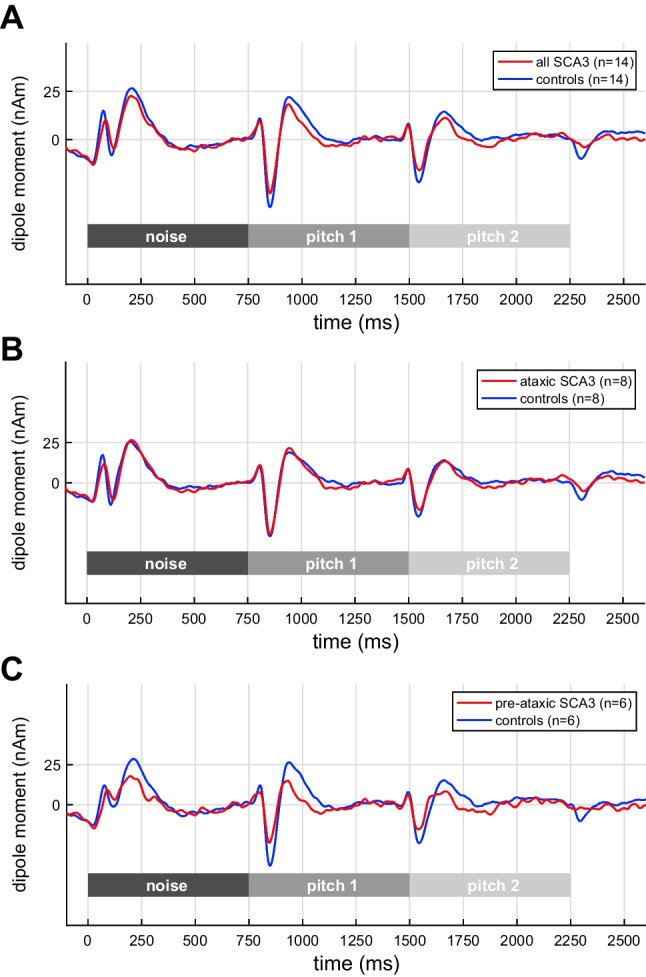
Grand-average MEG source waveforms, pooled across hemispheres, in response to pitch onset and pitch change in the IRN condition. **A** All SCA3 mutation carriers; **B** ataxic: ataxic SCA3 mutation carriers; **C** pre-ataxic: pre-ataxic SCA3 mutation carriers

Figure 2 shows the grand-average MEG source waveforms, pooled across hemispheres, in response to pitch onset and pitch change in the IRN condition. The onset of noise from silence elicited the typical tripartite P1-N1-P2 complex with comparable morphology between the groups; then, the pitch onset following the noise-IRN transition evoked a strong N100m wave (POR). As expected, the magnitude of the IRN-related POR was not different between groups: POR decrements occurred neither for the comparison of the whole sample (Fig. 2A: *W* = 74, *p* = 0.1426) nor for the sub-groups of ataxic (Fig. 2B: *W* = 30, *p* = 0.4392) or pre-ataxic SCA3 mutation carriers (Fig. 2C: *W* = 9, *p* = 0.0898). Subsequently, the transition from the first to the second (different) IRN pitch evoked another N100m wave (PCR) which was also not different between groups (whole group: *W* = 72, *p* = 0.1228; ataxic patients: *W* = 27, *p* = 0.3227; pre-ataxic mutation carriers: *W* = 10, *p* = 0.1201).

**Fig. 3:**
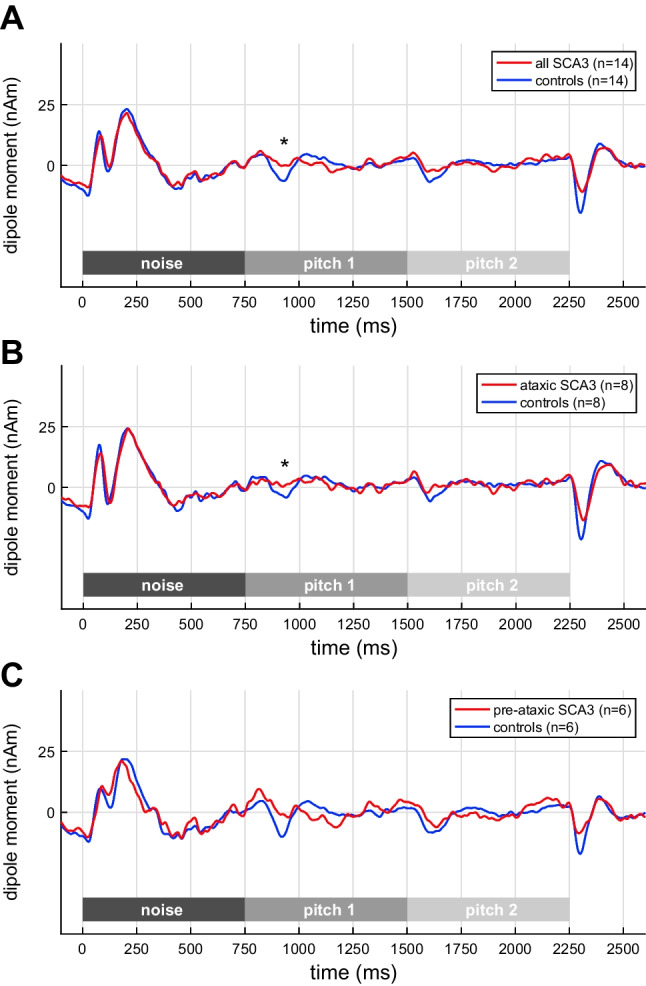
Grand-average MEG source waveforms, pooled across hemispheres, in response to pitch onset and pitch change in the Huggins pitch condition. **A** All SCA3 mutation carriers; **B** ataxic: ataxic SCA3 mutation carriers; **C** pre-ataxic: pre-ataxic SCA3 mutation carriers

Contrary to the (diotic) IRN condition, prominent between-group differences were found in the MEG responses to (binaural) Huggins pitch; the corresponding source waveforms are shown in Fig. 3, and they nicely reflect the result pattern of the psychoacoustic task. SCA3 mutation carriers exhibited a significantly attenuated N100m magnitude when compared to healthy controls (Fig. 3A: *W* = 54, *p* = 0.0222). The difference was mainly driven by the POR decrement in ataxic patients (Fig. 3B: *W* = 12, *p* = 0.0190), while the pre-ataxic SCA3 mutation carriers did not differ from the controls regarding their POR magnitude (Fig. 3C: *W* = 13, *p* = 0.2424).

**Fig. 4:**
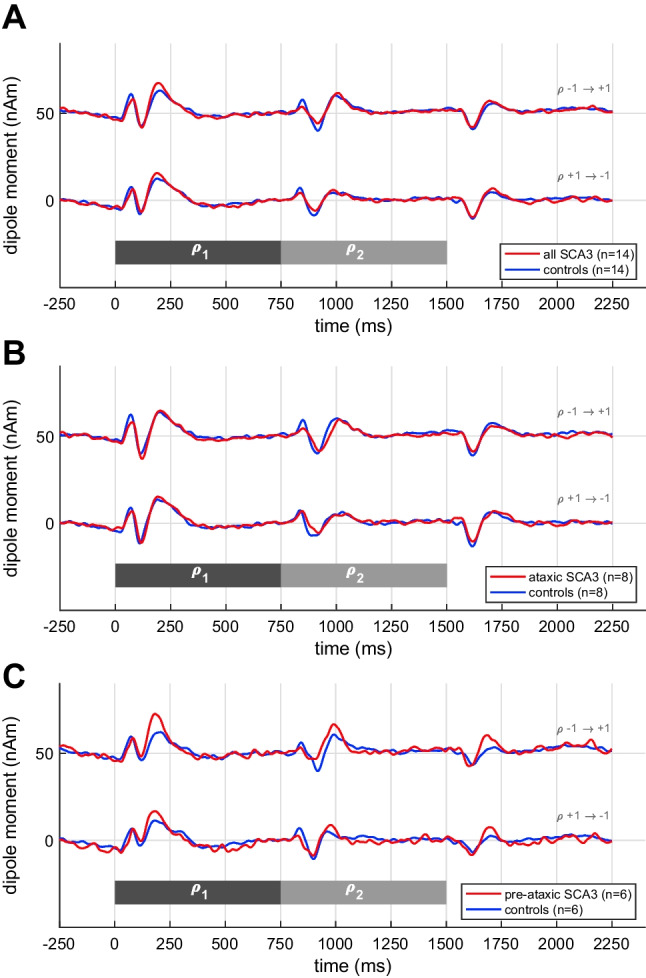
Grand-average MEG source waveforms, pooled across hemispheres, in response to changes in the interaural correlation of broadband noise. **A** All SCA3 mutation carriers; **B** ataxic: ataxic SCA3 mutation carriers; **C** pre-ataxic: pre-ataxic SCA3 mutation carriers

Figure 4 shows the source waveforms of the interaural correlation condition. Contrary to our expectations, changing the interaural correlation of the broadband noise did not induce significant N100m magnitude differences between groups, neither regarding the whole group (Fig. 4A: rho-mp: *W* = 66, *p* = 0.0794; rho-pm: *W* = 67, *p* = 0.0818) nor in ataxic patients (Fig. 4B: rho-mp: *W* = 26, *p* = 0.2869; rho-pm: *W* = 22, *p* = 0.1641) or in pre-ataxic SCA3 mutation carriers (Fig. 4C: rho-mp: *W* = 11, *p* = 0.1548; rho-pm: *W* = 13, *p* = 0.2424).

In a final analysis step, the source waveforms of the interaural correlation condition were used to screen for between-group differences in the EOR, i.e., the N100m response to the onset of the positively correlated (*r* = + 1) noise from silence. As expected, no such differences were found regarding this activity (all SCA3 mutation carriers: *W* = 67, *p* = 0.0819; ataxic patients: *W* = 22, *p* = 0.1641; pre-ataxic SCA3 mutation carriers: *W* = 13, *p* = 0.2424).

**Fig. 5:**
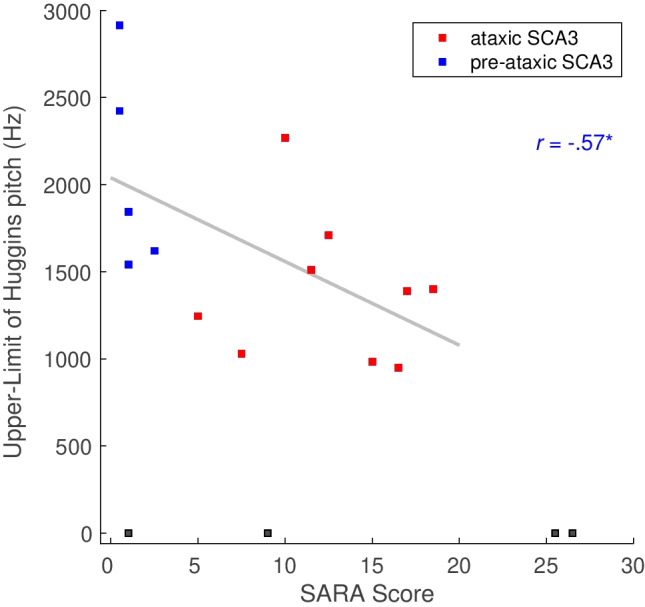
Correlation of SARA sum score with upper limit of Huggins pitch. Note that 4 SCA3 mutation carriers were not able to receive Huggins pitch at all

Further, we correlated the SARA sum score as a measure of ataxia severity with the upper limit of Huggins pitch and found a fairly strong correlation (*r* = − 0.57; Fig. 5).

## Discussion

The current study has shown, for the first time, that SCA3 mutation carriers differ from healthy, age-matched controls in their perceptual capability and neurophysiological representation of Huggins pitch. Specifically, the frequency limit up to which Huggins pitch could be heard was significantly lower among SCA3 mutation carriers, and their specific MEG response to Huggins pitch onset was diminished in magnitude. In the remainder of this paper, we discuss our findings in light of the existing literature and elaborate on some implications for future research and clinical assessment regarding these patients.

Our results are well in line with earlier reports of impaired binaural processing in SCA patients [8, 11]. The pons is known to play a central role in binaural synchronization along the ascending auditory pathway, and recent MRI studies have shown that this structure exhibits prominent tissue loss in SCA3 throughout the entire disease course [5, 6]. The present results indicate that Huggins pitch is a valuable tool to study the integrity of brainstem binaural synchronization and might perhaps even represent a candidate biomarker in SCA3 mutation carriers. Moreover, the experimental techniques underlying our findings are objective, quantitative methods that depend less than clinical scales on daily form or rater effects, and this benefit might foster future functional assessment in this group. Generally, psychoacoustic tests can be easily applied not only in specialized institutions but also at home if participants have the necessary equipment at hand (earphones, computer, quiet room) and do not wish to travel to an ataxia center located far away from their residence.

It is important to acknowledge that the SCA3-related effects in our data were driven by the subgroup of ataxic patients, whereas the pre-ataxic SCA3 mutation carriers had similar psychoacoustic thresholds and neuromagnetic responses like healthy controls. Difficulties with Huggins pitch appear to reflect impairment in SCA3 patients when ataxic symptoms are already present, but they do not allow to uncover pre-ataxic functional changes. Since Faber et al. [2021] showed that brainstem atrophy can already be detected in the in pre-ataxic stage of SCA3 mutation carriers, our aim is to carefully refine the experimental parameters of binaural stimuli such that more subtle processing alterations can be accessed earlier in disease course. As a second cautionary note, the current results arise from a cross-sectional proof-of-concept study, and at this point we do not know how the perception and neural processing of Huggins pitch in SCA3 patients changes over time. However, we found a fairly good correlation between the SARA sum score as a measure of ataxia severity and the upper limit of Huggins pitch suggesting that this psychoacoustic measure which can be easily administered might be employed as a functional parameter of disease progression. Yet, psychoacoustic AFC tasks as well as cortical N100 measures are known to have good reliability [31, 32], and this provides the basis for longitudinal investigations.

The SCA3 mutation carriers in the present study were carefully age-matched with a control group of healthy adults. It is therefore unlikely that the perceptual and neuromagnetic decrements of this group regarding Huggins pitch simply occurred due to age-related changes in binaural processing [14]. Interestingly, the SCA3-related MEG decrement arose solely in the Huggins pitch condition but not in the condition that included an IRN pitch *without binaural* synchronization, and also not in the condition that included interaural broadband correlation changes *without pitch*. One might now infer that the SCA3-related MEG response decrement refers to a peculiar combination of the very two aspects “binaural” and “pitch.” However, we should keep in mind that Huggins pitch results from a phase shift in a narrow high-frequency band where microsecond differences between the left vs right ear must be precisely encoded for the subtle percept to arise at all, without any benefit from low-frequency cues (like in the broadband correlation condition). Thus, although it may be tempting to discuss the present results under the light of pitch perception, it is likely that the observed decrements in SCA3 patients arise from impaired binaural synchronization, *in*dependent of pitch-related temporal integration. [33, 34]

Having said that, the pitch feature in Huggins pitch has been proven to be suitable to assess brainstem-related dysfunction of binaural processing. Further improvements of the paradigm as well as the increase of the number of participants seem to be reasonable. In the present study, some SCA3 patients found it particularly hard to perceive Huggins pitch; i.e., they did not even detect the starting HP-*f*_0_ in the psychoacoustic task. At the same time, most pre-ataxic mutation carriers did not have more difficulties with Huggins pitch perception than healthy controls; so the question arises how to adapt the procedure in a manner that makes it easier to catch the percept *at all* and still yield higher discriminatory power. One solution might be to present Huggins pitch stimuli not in single-sound fashion but in the form of melodic contours. Changing HP-*f*_0_ within the melody could facilitate detection and tracking of the perceptual cue, but the paradigm would still allow to determine the existence region of Huggins pitch in individual listeners [35]. On the other hand, Huggins pitch perception might be impeded by changing the amount and/or the frequency bandwidth the phase shift in the left vs right ear; this could be a way to capture subtle impairments, e.g., in pre-ataxic SCA3 mutation carriers who already show structural alterations in the brainstem but had no difficulties with our task in its current version. A further approach for future research lies in the interplay between psychoacoustic and neuromagnetic correlates of Huggins pitch. In a subsequent experiment, it would be particularly interesting to combine both aspects individually for different listeners.

Previous work has reported evidence that cognitive functions related to hearing are influenced by neural activity arising in the cerebellum [36]. Such effects were not assessed within the current experiment; moreover, since pre-ataxic SCA3 mutation carriers (SARA < 3) also showed alterations in binaural hearing, one might simply argue that this binaural impairment exists independent of clinically manifest ataxia as a correlate of cerebellar dysfunction. Yet, cerebellar contributions to hearing certainly represent an important aspect which deserves explicit consideration in future studies on ataxia.

Finally, a cautionary note must be made regarding the effect of other peripheral hearing disorders on our results. All of our participants (SCA3 mutation carriers as well as age-matched controls) had self-reported normal hearing; however, since audiometric thresholds were not available, we cannot entirely rule out that sensorineural hearing loss might have contributed to the observed between-group differences. Generally, audiological impairments are not uncommon among SCA patients, especially alterations in brainstem auditory-evoked potentials [37, 38]; yet, it is important to note that the HP *f*_0_ values in our MEG experiment were clearly below the frequency region above which audiological alterations are typically seen in SCA patients [8] (cf. Table 4). Furthermore, the neuromagnetic response to IRN stimuli was not different between groups, suggesting that processing based on monaural temporal precision was not impaired in our SCA3 mutation carriers. Therefore, while some influence of other peripheral disorders on our results cannot be excluded, it appears reasonable to assume that this was not the driving factor with respect to HP processing difficulties in the SCA3 group.

## Conclusion

Our results show that binaural processing is a promising and easy-to-use parameter to study the functional integrity of the olive complex. In future studies, the specific combination of psychoacoustic, neurophysiological, and imaging findings could be used to gain a more complete understanding of SCA3 pathology on a functional and structural level. Furthermore, such multivariate observations could also be used to more precisely map the progression of pre- and post-onset ataxia in mutation carriers.

## Data Availability

Neurophysiological data is available in OSF (https://osf.io/rmshp/).
